# Anatomy and Histology of the Mouth and Teeth

**Published:** 1913-03-15

**Authors:** 


Anatomy and Histology of the Mouth and Teeth. A new and fourth edition of Broomell and Fischelis' well-known work on Dental Anatomy and Histology is just published by P. Blakiston's Sons & Co. The former editions are so well-known and appreciated by dental instructors that this work has become a general favorite. This edition will be found more helpful as much has been added in the chapters on blood, lymph, the circulatory apparatus and glandular structures, formerly this knowledge seemed of little importance to the dental student and was given slight consideration by most teachers of dental students. The importance of a thorough knowledge of these tissues and their function is necessary that the dentist may treat oral diseases associated with the teeth intelligently. The text with references for collateral reading and especially the illustrations, set forth this new matter in a clear and entertaining manner and we believe it will add much to the value of the work as a teaching manual. In schools where the subject of dental antomy and histology are taught by the same instructor there is no text book that will take the place of this one. It is a book also that should appeal to the practitioner, but unfortunately we have little hopes that the average practitioner will soon interest himself in a good dental library.

